# Genetic and nongenetic factors associated with the fate of maiden ewe lambs: slaughtered without ever lambing versus retained for breeding^1^

**DOI:** 10.1093/tas/txz156

**Published:** 2019-09-24

**Authors:** Noirin McHugh, Thierry Pabiou, Kevin McDermott, Eamon Wall, Donagh P Berry

**Affiliations:** 1 Teagasc, Animal & Grassland Research and Innovation Centre, Moorepark, Fermoy, County Cork, Ireland; 2 Sheep Ireland, Highfield House, Shinagh, Bandon, County Cork, Ireland

**Keywords:** efficiency, genetics, sheep, survival, weight

## Abstract

The decision on which ewe lamb to retain versus which to sell is likely to vary by producer based on personal preference. What is not known, however, is if any commonality exists among producers in the characteristics of ewe lambs that influence their eventual fate. The objective of the present study was to determine what genetic and nongenetic factors associate with the fate of maiden ewe lambs. The fate of each ewe lamb born in the present study was defined as either subsequently: 1) having lambed in the flock, or 2) was slaughtered without any recorded lambing event. A total of 9,705 ewe lamb records from 41 crossbred flocks were used. The logit of the odds of the ewe lamb being retained for lambing was modeled using logistic regression. Variance components were then estimated for the binary trait representing the fate of the ewe lamb using animal linear and threshold mixed models. The genetic correlations between fate of the ewe lamb and preweaning, weaning, or postweaning liveweight were also estimated. From the edited data set, 45% of ewe lambs born entered the mature flock as ewes. Ewe lambs reared as singles, with greater levels of heterosis but lower levels of recombination loss, born to dams that lambed for the first time as hoggets, with greater breed proportion of the Belclare, Suffolk, Texel, and Llyen breeds were more likely (*P* < 0.001) to eventually lamb in the flock than be slaughtered without ever lambing. Irrespective of the age of the animal when weighed, heavier ewe lambs were more likely to eventually lamb (*P* < 0.001). The genetic SD and direct heritability of fate of the ewe lamb estimated in the univariate linear model was 26.58 percentage units and 0.31 (SE = 0.03), respectively; the heritability was 0.30 when estimated using the threshold model. The corresponding direct heritability of fate of the ewe lamb estimated in the bivariate analyses with liveweight ranged from 0.29 (SE = 0.03; preweaning weight) to 0.35 (SE = 0.04; postweaning weight). The genetic correlations estimated between fate of the ewe lamb and the liveweight traits were weak to moderate but strengthened as the age of the ewe lamb at weighing increased. Results from this study provide an understanding of the factors producers consider when selecting females for retention versus slaughter which may form useful parameters in the development of a decision support tool to identify suitable ewe lambs for retention.

## INTRODUCTION

With a replacement rate of approximately 20% to 30% in many sheep populations (Wolfová et al., 2009; Bohan et al., 2019), a large number of surplus ewe lambs are produced annually. In Ireland, 70% of sheep farmers retain home-bred females as replacements (Bohan et al., 2017) which suggest that many females are sold off-farm either to other flocks or directly to an abattoir. The decision on which ewe lamb to retain versus which to sell is likely to vary by producer based on personal preference. What has not been established heretofore is if any commonality exists among producers in the characteristics of ewe lambs that influence their eventual fate.

There is a plethora of studies that have documented factors, like liveweight, to be associated with fertility traits, such as age at first calving or lambing, in both nulliparous cattle (Buskirk et al., 1995; Wathes et al., 2008; Brickell et al., 2009) and sheep (Jorgenson et al., 1993). Similarly, in an analysis of Irish dairy herds, Kelleher et al. (2016) reported that only 76% of what they deemed to be eligible dairy heifers actually calved. Such studies have, however, failed to investigate the reasons why such nulliparous animals did not subsequently enter the mature herd/flock. Hence, the objective of the present study was to investigate the genetic and nongenetic factors associated with the fate of maiden ewe lambs in an Irish sheep population. Results will be useful in firstly understanding the factors producers consider when selecting females for retention versus slaughter, but also as input parameters for a decision support tool in identifying suitable ewe lambs for retention; the latter could earmark females of preferred characteristics and sire line as potential candidates from which to select from, but could also complement other information available when selling lambs.

## MATERIALS AND METHODS

### Data

Data on a range of animal-specific events including date of birth, date of lambing, liveweight records, and slaughter information were extracted from the Irish national sheep database hosted by Sheep Ireland (http://www.sheep.ie). Since the data originated from a pre-existing database, it was not necessary to obtain animal care and use committee approval to conduct the study. Data were available on 43,419 nulliparous ewe lambs born in 112 flocks where slaughter and lambing data were recorded between the year 2010 and 2018, inclusive.

#### Fate of the nulliparous female.

The fate of each ewe lamb born was defined as either having subsequently: 1) lambed at least once in the flock, or 2) was slaughtered without ever having lambed. As the fate of ewe lambs born in 2018 was yet to be determined, only ewe lambs born between 2010 and 2017 were retained for analysis; 3,676 records were omitted. Ewe lambs that lambed at least once prior to 28 mo of age (irrespective of their fate thereafter) were coded as having lambed (McHugh et al., 2017). Ewe lambs that were slaughtered ≤365 d of age, and without a recorded lambing date, were coded as having been slaughtered. Ewe lambs with a slaughter or death date >365 d of age, or no recorded slaughter, death or lambing date prior to 28 mo of age, were removed; a total of 19,009 records were removed. Only flocks where ≥25% of ewe lambs were slaughtered within a given year were retained; 5,255 records from 144 flock-years were removed. Finally only ewe lambs that were retained in their birth flock until slaughter or first lambing were considered. Following edits, records from 15,394 nulliparae ewe lambs remained.

#### Rearing status.

Data were also available on the birth and rearing type of each ewe lamb. Birth type of the ewe lamb was defined as the recorded number of full-sib lambs (including the ewe lamb herself) born in the same litter as the ewe lamb (alive or dead); only birth types between one (singles) and four (quadruplets) were retained for analysis. Litter rearing size was defined as the number of lambs (including the ewe lamb herself) that was reared in the litter of the ewe lamb at day 7 postlambing; only litter rearing sizes between one and three were retained.

#### Liveweight.

Ewe lamb liveweight recorded at birth, preweaning, weaning, and postweaning were also available and were defined based on the editing criteria used in the national genetic evaluations (McHugh et al., 2017); 92% of ewe lambs had at least two liveweight records. For birth weight, only lambs weighed within the first 48 h after birth and weighing between 2 and 9 kg were retained. Preweaning liveweight was defined as the liveweight taken between 20 and 65 d of age; only records of lambs weighing between 12 and 32 kg were retained. Weaning weight was defined as the liveweight recorded between 66 and 120 d of age; only liveweight records between 20 and 55 kg were retained. Postweaning weight was defined as liveweight measured between 121 and 180 d of age; only liveweight records between 25 and 65 kg were considered. Muscle and fat depth were also measured when weighing the animal postweaning; only muscle depth measurements between 20 to 35 mm and fat depth measures between 1 to 15 mm were retained. Average daily gain (ADG) between consecutive liveweight records was calculated for each lamb; only calculated ADG values between 100 and 650 g/d were retained which resulted in 1,054 ADG records being discarded.

Data were also available on dam parity, breed composition, and both heterosis and recombination loss coefficients of each ewe lamb and dam. Dam parity was categorized as 1, 2, 3, 4, 5, 6, or ≥7. Age of the dam at first lambing was categorized as lambing either: 1) between 8 and 18 mo of age, or 2) between ≥18 and 28 mo of age. The breed proportion of each ewe lamb and dam for the five major performance recording breeds in Ireland namely, Belclare, Charollais, Suffolk, Texel, and Vendeen were calculated. Heterosis and recombination loss coefficients were calculated for each ewe lamb as $1-\sum_{i=1}^{n}sire_{i}\;\cdot \;dam_{i}$ and $1-\sum_{i=1}^{n}\frac{sire_{i}^{2}+dam_{i}^{2}}{2}$, respectively, where sire_*i*_ and dam_*i*_ are the proportion of breed *i* in the sire and dam, respectively. Heterosis and recombination loss coefficients were subsequently grouped into distinct classes based on the frequency distribution of the respective coefficients. For heterosis four distinct classes were formed: less than 10%, 10% to 50%, 51% to 99%, and 100%. For recombination loss, two classes were formed: less than 10% and 10% to 50%.

Each ewe lamb was allocated to a contemporary group based on flock by month-year of birth. Only contemporary groups with some variation in the fate of the ewe lamb were retained for the analysis; 938 records from 12 flock-years were removed. Contemporary groups with less than 5 records were not considered further. Following all edits, 9,705 ewe lamb records from 41 flocks remained.

### Statistical Analyses

#### Phenotypic analysis.

The logit of the odds of the ewe lamb being retained on-farm as a ewe (i.e., subsequently lambed herself) was modeled using logistic regression in PROC GENMOD (SAS Inst. Inc., Cary, NC) while accounting for the binomial distribution of the errors. A multiple regression model was built up using stepwise forward-backward regression, including interactions of biological interest; the significance threshold for entry and exit of variables into/from the model was set at 5%. Factors considered for inclusion as fixed effects in the model were: birth type of the ewe lamb, rearing type of the ewe lamb, contemporary group, parity of dam, age at first lambing of dam, the breed proportion of each ewe lamb and dam for six breeds (Belclare, Charollais, Lleyn, Suffolk, Texel, and Vendeen) each fitted as separate covariates, and the heterosis and recombination loss classes of both the ewe lamb and her dam. Lamb liveweight at birth, preweaning, weaning, and postweaning, ultrasound fat and muscle depth, as well as ADG between the four weight time points were also considered individually in different iterations of the model.

#### Genetic analysis.

For the genetic analysis, only 7,571 ewe lambs who had both a sire and dam recorded were retained. Variance components were estimated for the fate of the ewe lamb using univariate animal linear mixed models fitted in ASReml (Gilmour et al., 2009). For comparison purposes, a univariate threshold model for fate of the ewe lamb was also fitted. For the fate of the ewe lamb, the fixed effects employed in all models were based on the results from the phenotypic analysis, except that lamb liveweight, ADG, or breed proportion of the ewe lamb or dam were not considered in the models. For both the linear univariate and threshold model, an additive direct animal effect was included as a random effect. Variance components estimated using a sire threshold model was almost identical to those estimated using the animal threshold model and are therefore not discussed. Including a maternal genetic or maternal permanent environmental effect did not improve the fit of the models. The pedigree of each animal was traced back to the founder population; breed groups were fitted via the pedigree.

A series of bivariate models were also investigated whereby ewe lambs with no known fate (i.e., had no recorded slaughter date or lambing date) that originated from the same flock-year as the ewe lambs with a known fate and had a recorded liveweight at either preweaning, weaning, or postweaning were re-introduced into the data set; 8,266 ewe lambs with liveweight records were added to give a total of 15,837 records. When the dependent variable was preweaning, weaning, or postweaning liveweight, the fixed effects included in the model were: age at weighing, contemporary group of flock-date of weighing, birth type of the ewe lamb, rearing type of the ewe lamb, dam parity, age of dam at first lambing, and ewe lamb and dam heterosis and recombination class. A direct additive genetic effect and maternal dam effect were both included as random effects for all liveweight traits.

## RESULTS

### Phenotypic Analysis

On average, 45% of the ewe lambs had a recorded lambing date, whereas the remaining 55% were slaughtered ≤365 d of age. The mean age at first lambing of ewe lambs was 611 (SD = 163) d; the median age at first lambing was 726 d. For the ewe lambs that were slaughtered, the mean (and median) age at slaughter was 226 (SD = 54) d.

Factors associated with the fate of the ewe lamb were rearing type, age of dam at first lambing, lamb heterosis and recombination class, dam heterosis class, the ewe lamb breed proportion for Belclare, Texel, and the Llyen breed as well as the dam breed proportion of the Belclare and Suffolk breed (*P* < 0.001); the fate of the ewe lamb did not differ by birth type, ewe parity, or class of recombination loss in the ewe lamb. Ewe lambs reared as singles were 1.37 times (*P* < 0.001) and 1.96 times (*P* < 0.001) more likely to eventually lamb compared to ewe lambs reared as twins and triplets, respectively. Ewe lambs born to dams that lambed for the first time as hoggets were 1.90 times (95% CI: 1.31 to 2.74; *P* < 0.001) more likely to, they themselves, subsequently lamb compared to ewe lambs born to dams that lambed for the first time as ewe lambs.

Ewe lambs with greater levels of heterosis were more likely to eventually lamb (Table 1); relative to ewe lambs with <10% heterosis, ewe lambs with 51% to 99%, and 100% were 1.75 times (*P* < 0.001) and 1.31 times (*P* < 0.05) more likely to eventually lamb, respectively (Table 1). Ewe lambs with lower levels of recombination loss (i.e., <10%) were 1.52 times (95% CI: 1.19 to 1.95; *P* < 0.001) more likely to eventually lamb relative to ewe lambs with 10% to 50% recombination loss. Ewe lambs born to dams with lower levels of heterosis were also more likely to eventually lamb (Table 1).

**Table 1.** Ewe lamb and dam heterosis and ewe lamb recombination odds ratios (upper and lower confidence intervals in parenthesis) for fate of ewe lamb

Trait	Level^1^	Odds ratio
Ewe lamb heterosis	<10%	1.00^a^
	10% to 50%	1.28 (0.93, 1.77)^a^
	51% to 99%	1.75 (1.26, 2.42)^b^
	100%	1.31 (0.99, 1.74)^a^
Dam heterosis	<10%	1.00^a^
	10% to 50%	0.69 (0.49, 0.99)^b^
	51% to 99%	0.73 (0.51, 1.04)^ab^
	100%	0.68 (0.53, 0.85)^b^
Ewe lamb recombination	<10%	1.00^a^
	10% to 50%	0.66 (0.51, 0.85)^b^

^1^Heterosis class of <10% was the reference category for ewe lamb and dam heterosis, and ewe lamb recombination.

^a,b^Least square means with different superscripts differ (*P* < 0.05) from each other.

The fate of the ewe lamb differed by the breed proportion of the Belclare, Suffolk, Texel, and Llyen in the ewe lamb herself. A ewe lamb of 50% Belclare breed proportion had a 13.0 percentage unit greater predicted probability of eventually lambing relative to a ewe lamb with a Belclare breed percentage of 25%. Ewe lambs born to dams with Belclare and Suffolk bloodlines had a lower probability of subsequently lambing; dams with a 50% breed percent of Belclare or Suffolk had a respective 4.19 percentage unit and 6.92 percentage unit lower predicted probability of producing ewe lambs who themselves eventually lambed compared to dams with 25% breed percent of the respective breeds.

**Figure 1.**
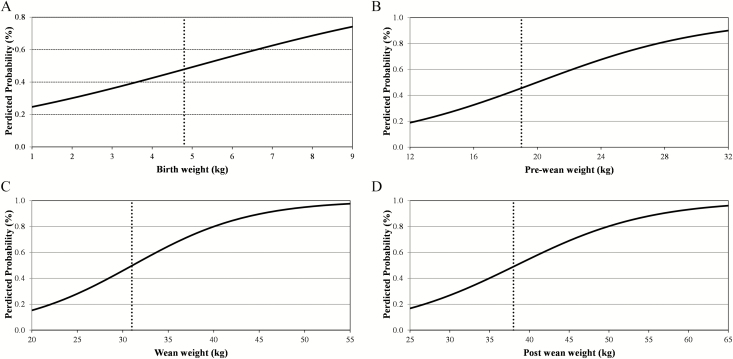
Predicted probability (continuous line) of eventually lambing for a ewe lamb differing in liveweight as well as the mean liveweight (dotted line) at (a) birth, (b) preweaning, (c) weaning, and (d) postwean^1^.

^1^Where the reference animal was a 50% Suffolk cross lamb reared as a twin, born to a 50% Belclare cross dam that lambed for the first time as a hogget, with heterosis of 10% to 50%, and recombination of 10% to 50%.

Irrespective of the time period of weighing, heavier ewe lambs were more likely to eventually lamb. A ewe lamb with a 1 SD heavier liveweight relative to the mean birth, preweaning, weaning, and postweaning weight had a 13.4, 30.7, 36.8, and 31.8 percentage unit greater predicted probability of eventually lambing compared to a ewe lamb that was 1 SD lighter than the mean liveweight at each respective time points (Fig. 1). When liveweight was included as the dependent variable and after adjusting for all of age at weighing, breed proportion, and birth and rearing type, ewe lambs that lambed were, on average, 0.27 kg (6% of the overall population mean), 1.68 kg (9% of the overall population mean), 3.21 kg (10% of the overall population mean), and 3.32 kg (9% of the overall population mean) heavier (*P* < 0.001) at birth, preweaning, weaning, and postweaning, respectively, compared to ewe lambs that were slaughtered at ≤365 d of age. Similarly ewe lambs that grew faster up to weaning were more likely (*P* < 0.001) to eventually lamb; the fate of the ewe lambs did not differ by ADG postweaning. Ewe lambs with greater fat and muscle depth recorded postweaning were more likely to eventually lamb; a ewe lamb with a fat depth of 7 mm (i.e., 1 SD higher than the mean fat depth) had a 7.9 percentage unit greater predicted probability of eventually lambing compared to a fat depth of 3 mm (1 SD lower than the mean fat depth). A ewe lamb with a muscle depth 1 SD deeper than the mean muscle depth (31 mm) had an 11.0 percentage unit greater predicted probability of eventually lambing compared to a ewe lamb with a fat depth 1 SD less than the mean muscle depth (25 mm; *P* < 0.001). The phenotypic factors associated with liveweight traits are presented elsewhere (McHugh et al., 2017) and are therefore not discussed.

### Genetic Analysis

The genetic SD and heritability of fate of the ewe lamb estimated from the linear univariate model was 26.58 percentage units and 0.31 (SE = 0.03), respectively (Table 2). The corresponding heritability of fate of the ewe lamb estimated using the threshold model was almost identical at 0.30 (SE = 0.01); therefore, only results from the linear model analysis of fate of the ewe lamb are presented hereon in. The direct heritability for the liveweight traits ranged from 0.10 (SE = 0.02) for preweaning liveweight to 0.27 (SE = 0.04) for postweaning liveweight. The maternal heritability for the liveweight traits reduced with lamb age from 0.24 (SE = 0.01) for birth weight to 0.07 (SE = 0.02) for postweaning weight. The genetic correlations estimated between fate of the ewe lamb and the liveweight traits were weak to moderate but strengthened as the age of the ewe lamb at weighing increased (Table 2). The corresponding phenotypic correlations between fate of the ewe lamb and the liveweight ranged from 0.16 (SE = 0.01) to 0.38 (SE = 0.01). The genetic SD for fate of the ewe lamb estimated in the bivariate analyses with liveweight measures ranged from 24.70 percentage units (weaning weight) to 26.50 percentage units (birth weight); the corresponding heritability of fate of the ewe lamb estimated in the bivariate analyses ranged from 0.29 (SE = 0.03; preweaning weight) to 0.35 (SE = 0.04; postweaning weight; Table 2).

**Table 2.** Direct genetic SD (σ _g_) in percentage units, the direct heritability for the fate of the ewe lamb estimated in a bivariate analyses with the liveweight traits as well as the corresponding genetic (*r*_g_; standard error in parentheses) and phenotypic (*r*_p_; standard error in parentheses) correlation

Trait	σ _g_	*h* ^2^_d_	*r*_g_	*r*_p_
Birth weight	0.265	0.31 (0.03)	0.22 (0.09)	0.16 (0.01)
Prewean	0.251	0.29 (0.03)	0.27 (0.09)	0.27 (0.01)
Wean	0.247	0.29 (0.03)	0.34 (0.08)	0.37 (0.01)
Postwean	0.256	0.35 (0.04)	0.41 (0.09)	0.38 (0.01)

## DISCUSSION

Intensive sheep production systems globally are typically characterized by large flock sizes with multiple different subflocks all being managed differently. This is particularly true prelambing where the number of in utero fetuses detected in mid-gestation is often used to partition the ewes into management groups based on expected litter size, with each group then fed accordingly. The same is true postlambing, where the management of ewes rearing multiple lambs often differs to those rearing singletons. Furthermore, if males remain entire, then the males may be managed separately to the female flock(s). Hence, the infrastructure and precedence exists to manage different subsets of the flock differently. While some producers may identify candidate replacement females at birth, others may not select females until later in life. In larger flock, such an exercise can become unwieldy. One strategy to facilitate the segregation of candidate ewe lambs for replacement (either for the flock itself or to market as replacements) would be to have a real-time decision support complemented with individual animal identification and genomic information; the latter could be used to estimate breed composition and parentage, enabling breeding values of the animal to be generated for the range of characteristics available. The extent of genotyping of candidate female replacements is intensifying in sheep as a means of more accurately identifying genetically elite females (Rupp et al., 2016). The net return from investing in genotyping females is a function of, among others, the proportion of genotyped females that are retained as replacements (Newton and Berry, 2019). This is because if fewer candidate females are selected, then the cost of genotyping the entire cohort of candidate females is diluted across the fewer number of selected females. If the candidate replacement females therefore could be prescreened based on factors identified in the present study, then fewer candidate females would need to be genotyped, implying a lower overall genotyping cost and thus reduced cost per retained female.

The fact that 45% of the females in the present study eventually re-entered the mature flock as replacements implies that there is indeed a huge opportunity to guide the decision on which females to retain. On the flipside, knowledge of the females which are certainly not suitable replacement candidates (i.e., low probability of suitability) can be extremely useful when partitioning out these females for either direct sale or for a management system suited for rapidly growing these females specifically for slaughter. The females could be further segregated on liveweight to produce a more homogenous group but also to advise on the appropriate feeding regime to achieve the desired drafting schedule.

### Risk Factors

The literature is generally void of factors associated with the fate of a young female sheep. The results from the present study indicate a greater likelihood of a female lamb becoming a replacement if she was born as a singleton, to a dam that lambed for the first time as a hogget (i.e., 2-yr old), had greater heterosis levels, and had greater ADG recorded preweaning. This is not overly surprising since these factors had been shown to associate with lamb liveweight (McHugh et al., 2017) and, with the exception of lamb breed composition, the factors associated with the likelihood of a female lamb becoming a replacement are based on the ewe lamb attaining a heavier liveweight at the point of selection. What may be surprising was the degree of the effect of some of the risk factors on ewe lamb fate and, in some cases, there was double the likelihood of being retained as a replacement when comparing some levels of the risk factors; examples include rearing type with almost twice the likelihood of a singleton being retained than a triplet or almost twice the likelihood of a ewe lamb being retained if born to a hogget compared to being born from a ewe lamb. A singleton ewe lamb, for example, born to a dam that lambed for the first time as a hogget, with 100% heterosis had a 31 percentage unit greater predicted probability of she herself eventually lambing in the flock compared to a ewe lamb born as a triplet to a dam that lambed for the first time as a ewe lamb with <10% heterosis. When lamb liveweight was added as a covariate to the model, the effect of some of the risk factors on ewe lamb fate was actually reversed; in fact, once adjusted to a common liveweight, ewe lambs reared as triplets and twins were 1.21 and 1.60 times (*P* < 0.001) more likely to eventually lamb relative to single-reared ewe lambs. This suggests that producers should segregate potential replacements females reared in multiple litters at a young age and ensure that sufficient feed resources are available to these ewe lambs to reach a desired liveweight prior to actual selection.

What the analysis undertaken in the present study, however fails to disentangle is the reasons why some females never subsequently lambed while others did. The outcome could be a conscious decision of the farmer not to even consider mating some females, but instead managing them for meat production, but could also be an inability of the female to conceive and establish pregnancy. In some instances of course, the slaughtered females may actually be pregnant. There is a paucity of information on conception rate in nulliparous sheep. However, in their analysis of 19 UK dairy herds, Wathes et al. (2008) stated that only 2.3% of heifers failed to conceive.

Notwithstanding these deficiencies, the large estimated genetic standard deviation for ewe lamb fate signifies an underlying genetic variability which could be considered in breeding programs. The existence of a moderate heritability (irrespective of statistical model) implies firstly that there is commonality among producers on the characteristics dictating the fate of the ewe lamb, and secondly that a large quantity of individual progeny data is not actually required to generate accurate estimates of genetic merit for individual sires. The similar heritability estimates for ewe lamb fate estimated using either a linear or threshold model is not unexpected given that the incidence of females in the present study that eventually reentered the mature flock as replacements was near 50% (i.e., 45%). Moreover, the moderate heritability of ewe lamb fate is similar to that for liveweight (Table 2; Safari et al., 2005) and higher than that generally reported for most fertility traits in sheep (Safari et al., 2005). This therefore implies that it is traits associated with animal size that influences farmer decisions on the fate of the ewe lamb as opposed to an inability to establish pregnancy; this conclusion is consistent with the observed genetic and phenotypic associations detected in the present study between animal liveweight and ewe lamb fate.

### Deployment of Decision Support Tool

A decision support tool developed using the parameters derived in the present study to identify candidate replacement females could be invoked at the time of each weighing with the candidate females diverted to a separate management group. At the subsequent weighing, selection could again occur within the candidate replacement group with some females possibly being ejected from the group; likewise, based on the performance data available at the time, females in the other mobs may graduate into the candidate replacement group. The model parameters estimated in the present study could form the basis of the suitability of each lamb as a flock replacement female. The predicted probability of the female being a suitable replacement could be calculated immediately with the available data used to populate the prediction equations generating the results. Ideally, the individual flock data would be used to generate the model parameters, although a large flock would be required to generate precise model solutions. A further option may be to use a Bayesian-type approach where the model solutions from larger data sets (e.g., the present data set) could act as prior knowledge which could then be combined with model solutions from the flock itself to generate a posterior probability for each lamb of becoming a replacement female.

## CONCLUSIONS

Despite an undoubted contribution of personal preferences to whether or not a ewe lamb is retained within the flock, results from the present study clearly indicate relatively strong consistency among Irish producers at least. The evidence of this was based on the often large odds ratios for some risk factors but also the moderate heritability for ewe lamb fate. Potential therefore exists for the development of a breeding and management decision support tool to inform and make both easier and automated the selection of females for retention as parents of the next generation in the flock.
